# Supplementary material for: Effect of human umbilical cord-derived mesenchymal stem cells on lung damage in severe COVID-19 patients: a randomized, double-blind, placebo-controlled phase 2 trial
Source: Signal Transduct Target Ther. 2021 Feb 10;6:58. doi: 10.1038/s41392-021-00488-5 (PMC7873662; doi:10.1038/s41392-021-00488-5)
Supplement: Supplementary file 1 — Supplementary Materials [file 41392_2021_488_MOESM1_ESM.pdf]

Supplementary Materials for

Effect of human umbilical cord-derived mesenchymal stem cells on lung damage in severe COVID-19 patients: a randomised, double-blind, placebo-controlled phase 2 trial

Lei Shi, Hai Huang, Xuechun Lu, Xiaoyan Yan, Xiaojing Jiang, Ruonan Xu, Siyu Wang, Chao Zhang, Xin Yuan, Zhe Xu, Lei Huang, Jun-Liang Fu, Yuanyuan Li, Yu Zhang, Wei-Qi Yao, Tianyi Liu, Jinwen Song, Liangliang Sun, Fan Yang, Xin Zhang, Bo Zhang, Ming Shi, Fanping Meng, Yanning Song, Yongpei Yu, Jiqiu Wen, Qi Li, Qing Mao, Markus Maeurer, Alimuddin Zumla, Chen Yao, Wei-Fen Xie, Fu-Sheng Wang

Correspondence to: fswang302@163.com, weifenxie@medmail.com.cn , yaochen@hsc.pku.edu.cn

This PDF file includes:

Figures S1 to S3

Tables S1 to S6

Data S1 to S5

Figure. S1. Protocol deviation and per-protocol population.

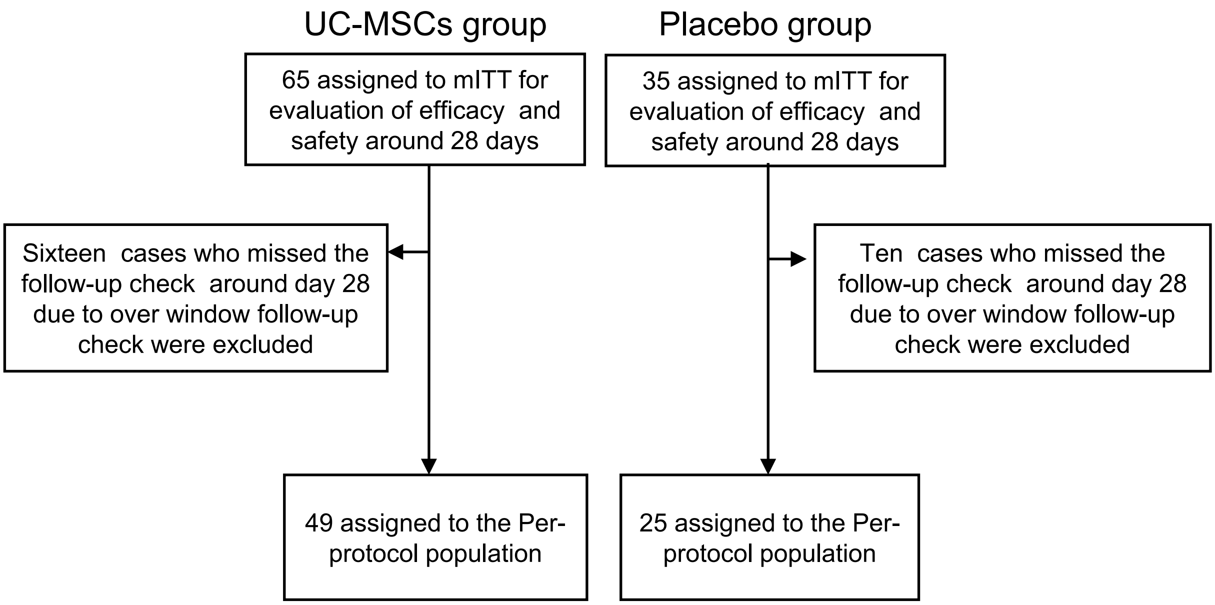

Figue S2. Dynamics of absolute counts of lymphocyte subsets and proinflammatory cytokines.

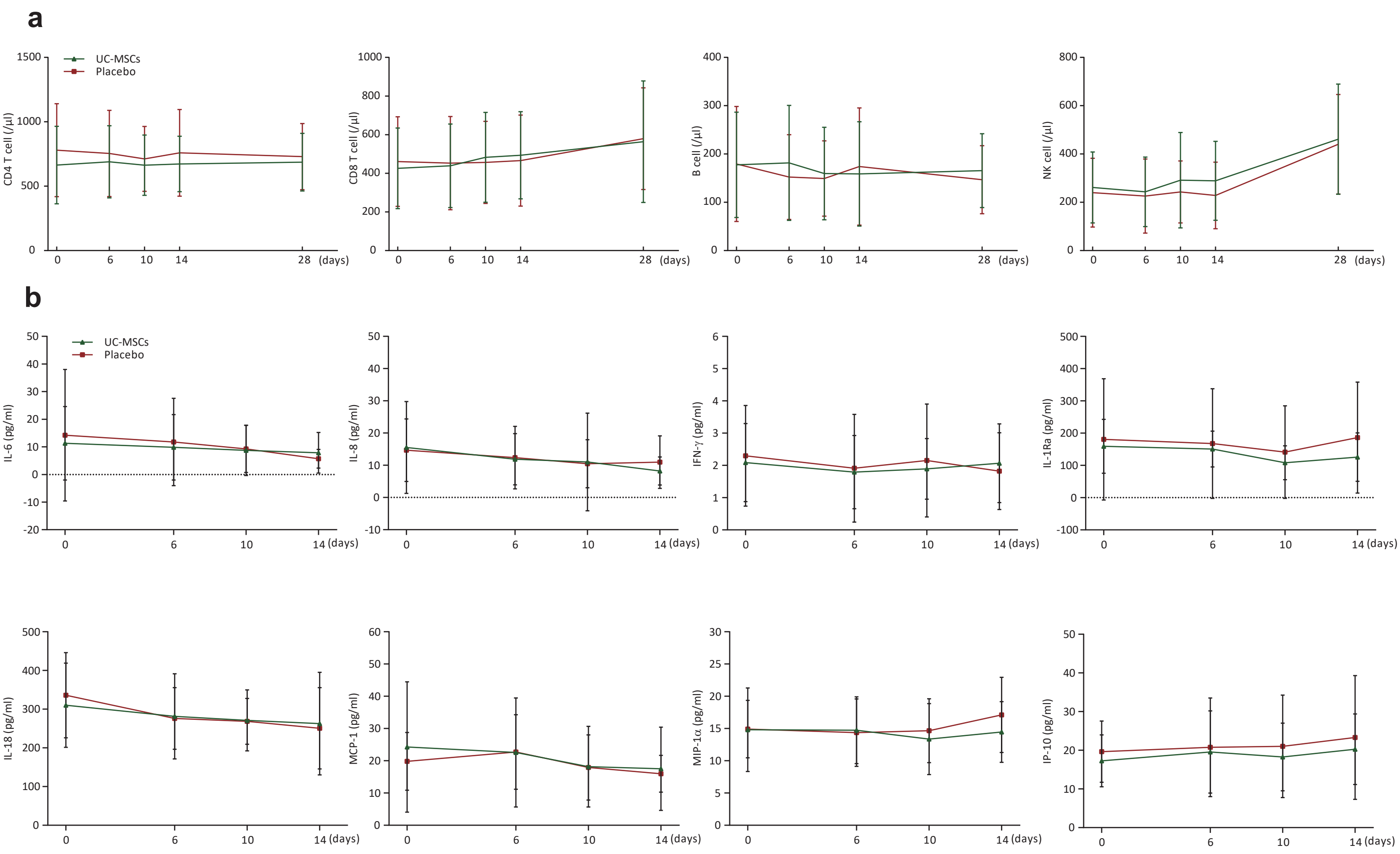

a. Absolute counts of CD4 T, CD8 T, B and NK cells at day 0, 6, 10, and 14 after infusion.
b. Plasma levels of IL-6, IL-8, IFN-γ, IL-1Ra, IL-18, MCP-1, MIP-1α, and IP-10 at day 0, 6, 10, and 14 after infusion.

Figure. S3. Presentative data of viable cell analysis of MSC product after its preparation in Tianjin and before intravenous transfusion in Wuhan.

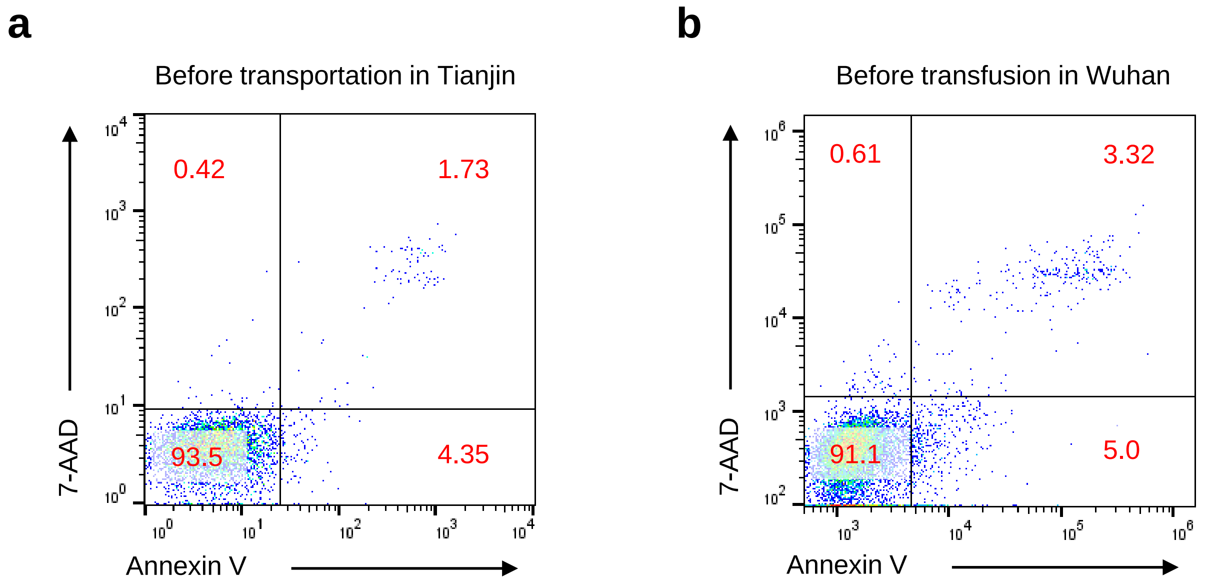

c

Date	0316	0318	0319	0320	0321	0322	0323	0324	0325	0326
**Tianjin**	93.9	95.0	93.9	95.3	94.7	94.7	93.3	96.8	89.6	96.3
**Wuhan**	87.8	87.9	79.8	92.2	90.6	93.1	84.9	93.4	80.9	93.4

a. Representative flow cytometric plots for vitality test of UC-MSCs before transportation in Tianjin using 7-AAD/ Annexin V assay.
b. Representative flow cytometric plots for vitality test of UC-MSCs before transfusion in Wuhan using 7-AAD/ Annexin V assay.
c. Detailed results of cell vitalities after preparation in Tianjin and upon arrival in Wuhan.

Table S1: Lesion proportion (%) of whole lung volume at day 0, 10, 28.

	Day	UC-MSCs group (n=65)	Placebo group (n=35)	Difference
**Total lesion** proportion (%) of whole lung volume	0	26.31(11.62,38.42)	27.98(11.57,44.14)	-1.91(-10.66,5.38) †
	10	25.99(6.32,39.95)	25.24(11.62,47.12)	-2.24(-10.65,5.38) †
	28	19.52(6.49,30.23)	17.21(9.03,38.25)	-1.77(-9.50,5.31) †
**Solid component lesion** proportion (%) of whole lung volume	0	2.59(0.69,5.20)	2.52(0.77,4.91)	-0.05(-1.08,0.92) †
	10	1.15(0.22,3.05)	1.40(0.51,2.64)	-0.25(-0.86,0.27) †
	28	0.94(0.22,2.25)	1.45(0.51,2.66)	-0.32(-0.97,0.14) †
**Ground-glass lesion** proportion (%) of whole lung volume	0	21.17(10.22,34.12)	24.95(9.40,35.46)	-2.47(-9.82,4.37) †
	10	23.01(6.11,36.90)	23.22(10.21,40.22)	-2.22(-9.67,4.71) †
	28	18.89(6.14,28.40)	15.15(8.61,34.37)	-1.60(-8.60,5.40) †

Data are expressed as median (interquartile range, IQR) The missing data has been imputed by LOCF(Last observation carried forward) strategy.

† Differences are expressed as Hodges-Lehmann estimator and 95% confidence interval (CI).

Table S2: Baseline patient characteristics in the Per-protocol population.

	UC-MSCs group (n=49)	Placebo group (n=25)
Age, years	60.43(9.26)	60.28(8.01)
Sex – no. (%)		
Men	27(55.10%)	13(52.00%)
Women	22(44.90%)	12(48.00%)
Body Weight, kilogram*	66.92(8.23)	66.35(8.04)
BMI (Body Mass Index), Kg/m^2*	24.89(3.14)	25.11(3.11)
Time from symptom onset to starting study treatment, days	45.00 (40.00,50.00)	49.00 (45.00,55.00)
Any comorbidities	25(51.02%)	13(52.00%)
Hypertension	13(26.53%)	7(28.00%)
Diabetes	9(18.37%)	3(12.00%)
Chronic bronchitis	2(4.08%)	2(8.00%)
Chronic obstructive pulmonary disease	2(4.08%)	0(0.00%)
Concomitant medication		
Antiviral drugs	23(46.94%)	15(60.00%)
Antibiotics	18(36.73%)	11(44.00%)
Glucocorticoid	9(18.37%)	6(24.00%)
Total lesion proportion (%): total lesion volume (in cm^3) / whole lung volume (in cm^3)	24.82(11.41,41.76)	27.28(12.16,45.32)
Solid component lesion proportion (%): Solid component lesion volume (in cm^3) / whole lung volume (in cm^3)	2.02(0.61,5.49)	2.20(0.76,4.61)
Six-category scale		
2-Hospitalized, not requiring supplemental oxygen	11(22.45%)	8(32.00%)
3-Hospitalized, requiring supplemental oxygen	37(75.51%)	17(68.00%)
4-Hospitalized, on noninvasive ventilation or high flow oxygen devices	1(2.04%)	0(0.00%)
White blood cell count (10^9/L)	5.70(5.20,6.60)	5.80(4.90,7.30)
Lymphocyte count (10^9/L)	1.39(1.19,1.80)	1.47(1.24,1.84)
CD4 T (/μl) †	643.00 (507.00,721.00)	808.00 (465.00,1169.50)

CD8 T (/μl) †	353.50 (275.00,484.00)	411.00 (306.50,576.50)
B (/μl) †	148.50 (112.00,215.00)	182.50 (107.15,263.50)
NK (/μl) †	198.00 (151.00,395.00)	220.00 (133.00,337.50)
Neutrophil count (10^9/L)	3.48(2.91,4.32)	3.83(2.85,4.48)
Platelet count (10^9/L)	214.00(174.00,255.00)	210.00(176.00,247.00)
Hemoglobin （g/L）	123.82(14.64)	122.92(11.29)
D-dimer (mg/L) ‡	0.58(0.36,1.10)	0.60(0.37,1.29)
IL-6 (pg/mL) §	7.63 (6.08,9.78)	8.76 (6.54,11.77)
CRP (mg/L) ¶	2.11 (1.16,3.47)	1.38 (0.68,2.14)
SARS-CoV-2 test result‖		
SARS-Cov-2 IgG positive	48(100.00%)	24(100.00%)
SARS-Cov-2 IgM positive	44(91.67%)	22(91.67%)
SARS-Cov-2 nucleic acid detection positive	36(73.47%)	14(56.00%)

Data are median (interquartile range (IQR)), n (%), ~~n/N(%),~~ or mean (SD)

* BMI values were available for 49 patients in the UC-MSCs group and 23 patients in the placebo group.

† CD4、CD8、CD19、CD56values were available for 46 patients in the UC-MSCs group and 24 patients in the placebo group.

‡ D-dimer values were available for 20 patients in the UC-MSCs group and 20 patients in the placebo group.

§ IL-6 values were available for 48 patients in the UC-MSCs group and 25 patients in the placebo group.

¶ CRP values were available for 21 patients in the UC-MSCs group and 10 patients in the placebo group.

‖ The test results are summarized from the hospitalization to the pre-random test. If there is any positive, it is defined as positive. . The IgG and IgM values were available for 48 patients in the UC-MSCs group and 24 patients in the placebo group.

Table S3: Imaging outcomes in the per-protocol population.

	UC-MSCs group (n=49)	Placebo group (n=25)	Difference
Change in **total** lesion proportion (%) of whole lung volume from baseline to day 28*	-19.32(-54.56,-3.74)	-8.13(-49.10,6.79)	-9.82(-29.26,9.41)
Change in **solid component** lesion proportion (%) of whole lung volume from baseline to day 28*	-54.29 (-73.44,-33.37)	-42.78 (-61.73,19.67)	-17.55(-41.04,1.88)
Change in **ground-glass** lesion proportion (%) of whole lung volume from baseline to day 28*	-18.46 (-57.32,5.61)	-6.30 (-45.69,8.05)	-6.39(-27.56,15.82)

*Lesion proportion = lesion volume (in cm^3) / full lung volume (in cm^3). Data are median (interquartile range, IQR).

† Differences are expressed as Hodges-Lehmann estimator and 95% confidence interval (CI).

Table S4. Analysis results of imaging outcomes for mITT based on the use of five models.

Model	UC-MSC group	Placebo group	Difference
Change in the total lesion proportion (%) of the whole lung volume from baseline to day 28			
Model1	-23.45(-33.24,-13.66)	-13.59(-26.93,-0.25)	-9.86(-26.41,6.68)
Model2	-23.39(-33.23,-13.54)	-13.55(-26.95,-0.14)	-9.84(-26.46,6.78)
Model3	-23.34(-33.22,-13.46)	-13.71(-27.18,-0.25)	-9.63(-26.33,7.07)
Model4	-23.45(-33.24,-13.66)	-13.59(-26.93,-0.24)	-9.86(-26.41,6.68)
Model5	-23.36(-33.19,-13.54)	-13.74(-27.13,-0.35)	-9.62(-26.24,6.99)
Change in solid component lesion proportion (%) of whole lung volume from baseline to day 28			
Model1	-47.69(-61.50,-33.89)	-21.83(-40.64,-3.03)	-25.86(-49.19,-2.53)
Model2	-47.47(-61.30,-33.64)	-21.69(-40.53,-2.86)	-25.77(-49.14,-2.41)
Model3	-47.47(-61.37,-33.56)	-21.70(-40.65,-2.75)	-25.77(-49.27,-2.26)
Model4	-47.69(-61.50,-33.89)	-21.83(-40.64,-3.02)	-25.86(-49.19,-2.53)
Model5	-47.65(-61.52,-33.77)	-21.92(-40.84,-3.00)	-25.73(-49.20,-2.26)
Change in ground-glass lesion proportion (%) of whole lung volume from baseline to day 28			
Model1	-14.80(-26.49,-3.10)	-7.73(-23.67,8.21)	-7.07(-26.84,12.70)
Model2	-14.70(-26.46,-2.95)	-7.67(-23.68,8.34)	-7.03(-26.89,12.82)
Model3	-14.68(-26.50,-2.87)	-7.74(-23.85,8.36)	-6.94(-26.92,13.04)
Model4	-14.80(-26.49,-3.10)	-7.73(-23.67,8.21)	-7.07(-26.84,12.70)
Model5	-14.75(-26.50,-2.99)	-7.82(-23.85,8.21)	-6.93(-26.81,12.96)

The above analysis models are based on covariance model.

Treatment group factor was included in model 1, treatment group and center factors were included in model 2 as fixed effects, and treatment group, baseline and center factors were included as fixed effects in model 3. Center factor was included as random effect, treatment group factor was included as fixed effects in model 4, center factor was included as random effect, treatment group and baseline factors were included as fixed effects in model 5.

Table S5. Analysis results of imaging outcomes for PPS based on the use of five models.

Model	UC-MSC group	Placebo group	Difference
Change in the total lesion proportion (%) of the whole lung volume from baseline to day 28			
Model1	-27.12(-37.82,-16.42)	-17.55(-32.68,-2.42)	-9.58(-28.11,8.96)
Model2	-27.04(-37.81,-16.27)	-17.38(-32.62,-2.14)	-9.66(-28.31,8.99)
Model3	-26.92(-37.66,-16.18)	-17.88(-33.09,-2.67)	-9.04(-27.66,9.58)
Model4	-27.12(-37.83,-16.42)	-17.55(-32.68,-2.41)	-9.58(-28.11,8.96)
Model5	-26.93(-37.59,-16.28)	-17.93(-33.00,-2.85)	-9.01(-27.48,9.46)
Change in solid component lesion proportion (%) of whole lung volume from baseline to day 28			
Model1	-44.28(-62.04,-26.52)	-10.09(-35.21,15.03)	-34.19(-64.96,-3.42)
Model2	-44.02(-61.83,-26.21)	-9.57(-34.77,15.64)	-34.45(-65.29,-3.62)
Model3	-43.97(-61.91,-26.03)	-9.77(-35.18,15.65)	-34.20(-65.30,-3.10)
Model4	-44.28(-62.05,-26.51)	-10.09(-35.22,15.04)	-34.19(-64.96,-3.41)
Model5	-44.15(-62.03,-26.28)	-10.35(-35.63,14.94)	-33.81(-64.78,-2.83)
Change in ground-glass lesion proportion (%) of whole lung volume from baseline to day 28			
Model1	-19.68(-32.18,-7.19)	-15.00(-32.67,2.67)	-4.68(-26.33,16.96)
Model2	-19.56(-32.13,-6.99)	-14.76(-32.54,3.03)	-4.81(-26.57,16.96)
Model3	-19.46(-32.06,-6.86)	-15.17(-33.02,2.68)	-4.29(-26.14,17.55)
Model4	-19.68(-32.18,-7.18)	-15.00(-32.68,2.68)	-4.68(-26.34,16.97)
Model5	-19.52(-32.03,-7.01)	-15.34(-33.04,2.36)	-4.18(-25.87,17.51)

The above analysis models are based on covariance model.

Treatment group factor was included in model 1, treatment group and center factors were included in model 2 as fixed effects, and treatment group, baseline and center factors were included as fixed effects in model 3. Center factor was included as random effect, treatment group factor was included as fixed effects in model 4, center factor was included as random effect, treatment group and baseline factors were included as fixed effects in model 5.

Table S6. Analysis results of imaging outcomes for ITT based on the use of five models.

Model	UC-MSC group	Placebo group	Difference
Change in the total lesion proportion (%) of the whole lung volume from baseline to day 28			
Model1	-23.09(-32.78,-13.41)	-13.59(-26.88,-0.29)	-9.51(-25.95,6.94)
Model2	-23.05(-32.77,-13.32)	-13.54(-26.89,-0.19)	-9.51(-26.02,7.01)
Model3	-22.99(-32.75,-13.23)	-13.70(-27.11,-0.29)	-9.29(-25.88,7.30)
Model4	-23.09(-32.78,-13.41)	-13.59(-26.88,-0.29)	-9.51(-25.96,6.94)
Model5	-23.01(-32.72,-13.30)	-13.74(-27.09,-0.40)	-9.27(-25.78,7.24)
Change in solid component lesion proportion (%) of whole lung volume from baseline to day 28			
Model1	-46.97(-60.65,-33.30)	-21.83(-40.61,-3.06)	-25.14(-48.37,-1.91)
Model2	-46.81(-60.50,-33.12)	-21.68(-40.47,-2.89)	-25.13(-48.37,-1.88)
Model3	-46.81(-60.57,-33.05)	-21.68(-40.59,-2.77)	-25.13(-48.52,-1.74)
Model4	-46.97(-60.65,-33.29)	-21.83(-40.62,-3.05)	-25.14(-48.37,-1.90)
Model5	-46.93(-60.67,-33.18)	-21.92(-40.81,-3.04)	-25.00(-48.37,-1.64)
Change in ground-glass lesion proportion (%) of whole lung volume from baseline to day 28			
Model1	-14.57(-26.12,-3.02)	-7.73(-23.59,8.14)	-6.84(-26.47,12.78)
Model2	-14.51(-26.11,-2.90)	-7.67(-23.60,8.27)	-6.84(-26.55,12.87)
Model3	-14.48(-26.15,-2.82)	-7.74(-23.76,8.29)	-6.75(-26.57,13.08)
Model4	-14.57(-26.13,-3.02)	-7.73(-23.59,8.14)	-6.84(-26.47,12.78)
Model5	-14.52(-26.14,-2.91)	-7.82(-23.77,8.13)	-6.70(-26.44,13.03)

The above analysis models are based on covariance model.

Treatment group factor was included in model 1, treatment group and center factors were included in model 2 as fixed effects, and treatment group, baseline and center factors were included as fixed effects in model 3. Center factor was included as random effect, treatment group factor was included as fixed effects in model 4, center factor was included as random effect, treatment group and baseline factors were included as fixed effects in model 5.

Data S1

A phase II, multicenter, randomized, double-blind, placebo-controlled trial to evaluate the efficacy and safety of human umbilical cord-derived mesenchymal stem cells in the treatment of severe COVID-19 patients

Protocol

Version 3.1 - June 1, 2020

Sponsor:	The Fifth Medical Center of PLA General Hospital
Principal investigator:	Fu-Sheng Wang
Ethics Reference Number:	2020-013-D
ClinicalTrials.gov	NCT04288102

Table of Contents

1. TRIAL SUMMARY 4

2. INTRODUCTION 8

2.1. Background and rationale 8

2.2. Objectives 8

2.3. Trial design 8

3. METHODS 9

3.1. Study setting 9

3.2. Eligibility criteria 9

3.2.1. Inclusion criteria 9

3.2.2. Exclusion criteria 9

3.3. Interventions 10

3.3.1. Investigational medicinal product 10

3.3.2. Standard of care (SOC) 10

3.3.3. Intervention assignment 10

3.3.4. Modifications of intervention 11

3.4. Outcomes 11

3.4.1 Imaging Endpoints by centralized interpretation 11

3.4.2 Other secondary endpoints 11

3.4.3 Safety endpoints 12

3.4.4 Centralized imaging interpretation of endpoints based on high-resolution chest computed tomography imaging 12

3.5. Participant timeline 14

3.6. Sample size 16

3.7. Recruitment 16

3.8. Allocation, blinding and product management 16

3.8.1. Allocation and concealment of allocation 16

3.8.2. Blinding 16

3.8.3. Product management 17

3.9. Data collection and data management 17

3.10. Statistical consideration 17

3.10.1. Statistical methods 18

3.10.2. Additional analyses 18

3.10.3. Analysis population and missing data 18

3.11. Safety/harms 19

3.12. Auditing 19

4. ETHICS AND DISSEMINATION 20

4.1. Research ethics approval.. 20
4.2. Protocol amendments.. 20
4.3. Informed consent process 20
4.4. Confidentiality 20
4.5. Declaration of interests 20
4.6. Access to data 20
4.7. Ancillary and post-trial care... 20
4.8. Dissemination policy 20
5. STUDY ADMINISTRATION.. 21
5.1. Key contacts.. 21
5.2. Roles and responsibilities 21
5.2.1. Protocol contributors... 21
5.2.2. Sponsor and Funding and Collaborators.. 21
5.2.3. Trial committees 22
6. References.. 22
7. APPENDICES 23
7.1 Revision History 24
7.2 mMRC (Modified Medical Research Council) Dyspnea Scale 28

1. TRIAL SUMMARY

World Health Organization Registration Data Set

Title	A phase II, multicenter, randomized, double-blind, placebo-controlled trial to evaluate the efficacy and safety of human umbilical cord-derived mesenchymal stem cells in the treatment of severe COVID-19 patients
Primary registry and trial identifying number	ClinicalTrials.gov NCT04288102
Secondary identifying numbers	2020-013-D
Sources of monetary or material support	The Fifth Medical Center of PLA General Hospital
Primary sponsor	The Fifth Medical Center of PLA General Hospital
Central contact	Principal Investigator: Fu-Sheng Wang, MD, Ph.D.<br>86-10-66933332 fswang302@163.com<br><br>Investigator and coordinator: Lei Shi, MD, Ph.D.<br>86-10-66933333 shilei302@126.com<br><br>Study Sites:<br><br>Wuhan Huoshenshan Hospital, Wuhan, Hubei, China, 430000<br>Contact: Lei Shi, MD, PhD<br><br>Optical Valley Branch of Maternal and Child Hospital of Hubei Province, Wuhan, Hubei, China, 430000<br>Contact: Wei-Fen Xie, MD, PhD<br><br>General Hospital of Central Theater Command (Follow-up only), Hubei, China, 430000<br>Contact: Xiaojing Jiang, MD, PhD
Study officials/Investigators	Study Principal Investigator<br>Fu-Sheng Wang, MD, PhD<br>The Fifth Medical Center of PLA General Hospital
Brief title	Treatment with human umbilical cord-derived mesenchymal stem cells for severe corona virus disease 2019 (COVID-19)

Countries of recruitment	P. R. China
Condition(s) or focus of study	COVID-19
Interventions	Human umbilical cord-derived mesenchymal stem cells (UC-MSCs)
Key eligibility criteria	**Inclusion criteria**<br>1. Age≥18 and <75 years;<br>2. hospitalized;<br>3. Severe COVID-19:<br>1) Laboratory confirmation of SARS-CoV-2 infection by reverse-transcription polymerase chain reaction (RT-PCR) from any diagnostic sampling source<br>2) Confirmed pneumonia by chest computed tomography imaging<br>3) Apply to any one of the following: 1)dyspnea (RR ≥ 30 times/min), 2)finger oxygen saturation ≤ 93% in resting state, 3)arterial oxygen partial pressure (PaO2) / oxygen absorption concentration (FiO2) ≤ 300MMHG, 4)pulmonary imaging shows that the focus progress > 50% in 24-48 hours<br>4. Confirmed interstitial lung damage by chest computed tomography imaging.<br><br>**Exclusion criteria**<br>1. Pregnancy, lactation and those who are not pregnant but do not take effective contraceptives measures;<br>2. Patients with malignant tumor, other serious systemic diseases and psychosis;<br>3. Patients who are participating in other clinical trials;<br>4. Inability to provide informed consent or to comply with test requirements.<br>5. Co-Infection of HIV, tuberculosis, influenza virus, adenovirus and other respiratory infection virus.<br>6. Invasive ventilation<br>7. Shock<br>8. Combined with other organ failure(need organ support)<br>9. Interstitial lung damage caused by other reasons (in 2 weeks)

	10. The pulmonary imaging revealed the interstitial damage of lungs before the COVID-19 confirmed.
Study design	Study type: Interventional trial<br>Allocation: Randomized<br>Intervention model: Parallel group<br>Primary purpose: Treatment<br>Phase: Phase 2
Masking	Double-blind
Date of enrollment	March 5, 2020.
Target sample size	90 (60 in UC-MSCs group, 30 in placebo group)
Recruitment status	Recruiting
Primary outcomes	**Primary endpoint measures (**centralized imaging interpretation**)**<br>• Change in lesion proportion (%) of full lung volume from baseline to day 28.<br>Lesion proportion = lesion volume (in cm^3) / full lung volume (in cm^3)
Secondary outcomes	**Secondary endpoint measures (**centralized imaging interpretation**)**<br>• Change in lesion proportion (%) of full lung volume from baseline to day 10 and 90<br>• Change in solid component lesion proportion (%) of full lung volume from baseline to day 10, 28 and 90.<br>• Change in ground-glass lesion proportion (%) of full lung volume from baseline to day 10, 28 and 90.<br>• Pulmonary fibrosis – related morphological features in CT scan at day 90<br>a. cord-like shadow<br>b. honeycomb-like shadows<br>c. interlobular septal thickening<br>d. intralobular interstitial thickening<br>e. pleural thickening<br>• Lung densitometry:<br>a. Change in total voxel ‘weight’ in lesion area from baseline to day 10, 28 and 90.<br>voxel ‘weight’=voxel density (in HU) × voxel volume (in voxel)

b. volumes histogram of lung density distribution (<-750, -750~-300, -300~50, >50) at day 10, 28 and 90.

Other secondary endpoints

- Time to clinical improvement in 28 days.

Clinical improvement defined as a one-point deduction from baseline in a 6 ordinal scale:

1. Not hospitalized;
2. Hospitalized, not requiring supplemental oxygen;
3. Hospitalized, requiring supplemental oxygen;
4. Hospitalized, on non-invasive ventilation or high flow oxygen devices;
5. Hospitalized, on invasive mechanical ventilation or ECMO;
6. Death.

- Change in oxygenation index (PaO2/FiO2) from baseline to day 6, 10, and 28.
- The duration of oxygen therapy (in days)
- Change in oxygen saturation from baseline to day 6, 10 and 28
- 6-minute walk test at day 28 and 90 (in meters)
- Pulmonary function test at day 28 and 90
  a. maximum vital capacity (VC_{max})
  b. diffusing Capacity (DL_{CO})
- Difference in the change of mMRC (Modified Medical Research Council) Dyspnea Scale at day 28, day 90.
- Changes of absolute lymphocyte counts and subsets, as well as cytokine/chemokine levels from baseline to day 6, 10, 28 and 90.

2. INTRODUCTION

2.1. Background and rationale

The Corona Virus Disease 2019 (COVID-19) caused by severe acute respiratory syndrome corona virus 2 (SARS-CoV-2) infection has unprecedentedly spread in the worldwide and been declared as a pandemic by the world health organization. COVID-19 is characterized by sustained cytokines production and hyper-inflammation, can cause clusters of severe respiratory illness with a fatality rate around 2-5%. There are currently no prophylactic vaccine and no specific antiviral treatment agents available recommended for COVID-19. The management of COVID-19 patients remains largely symptomatic and supportive therapy for severe patients. Therefore, it is urgent to find a safe and effective therapeutic approach to COVID-19.

During the last decade, the promising features of mesenchymal stem cells (MSCs), including their regenerative properties and ability to differentiate into diverse cell lineages, have generated great interest among researchers whose work has offered intriguing perspectives on cell-based therapies for various diseases. MSCs have already secured conditional approved for the treatment of children with graft-versus-host disease for its inflammation suppressing previlege. MSCs could significantly reduce the pathological changes of lung and inhibit the cell-mediated immune inflammatory response induced by influenza virus in animal model and clinical trial. In previous study, the safety and primary efficacy of MSC have been evaluated in patients with acute respiratory distress syndrome (ARDS) and the underlying mechanisms have been studied in some pre-clinical settings. It seems that MSC-based treatments principally contributed to inhibit the uncontrolled immune inflammatory response, benefit the recovery of lung function, and were conductive to delay the progress of lung fibrosis. Accordingly, these findings raise the hypothesis that use of MSCs transfusion could be beneficial in COVID-19 patients.

As a first step in testing safety, we did a phase 1, open-label trial in 20 patients with severe COVID-19, using a dose of 3×10^7 umbilical cord-MSCs(UC-MSCs) at 3-day intervals by intravenous reinfusion that may contribute to alleviate hyper-inflammation response. Infusions were well tolerated. Given the in-vitro and in-vivo benefit of MSCs and the acceptable tolerance and safety in humans, we developed this trial with the objective of evaluating the safety and efficacy of intravenous UC-MSCs in adults with severe pneumonia caused by SARS-CoV-2.

2.2. Objectives

To evaluate the efficacy and safety of umbilical cord-derived human mesenchymal stem cells in the treatment of severe COVID-19 patients.

2.3. Trial design

This trial is designed as a phase II, multi-center, randomized, double-blind, placebo-controlled trial. The allocation ratio is 2:1 in the testing group comparing to the control group. This is an investigator-initiated trial.

3. METHODS

3.1. Study setting

The trial will be conducted in two COVID-19 designated hospitals in Wuhan City, Hubei Province: Huoshenshan Hospital and Hubei Maternity and Child Health Care Hospital. Huoshenshan Hospital is a new-built hospital for this outbreak with 1000 beds capacity, and Hubei Maternity and Child Health Care Hospital is temporarily reformed for admitting COVID-19 patients from an under constructed specialized hospital with 800 beds capacity. After patients discharged from the COVID-19 designated hospitals, the follow-up procedure will be conducted at the General Hospital of Central Theater Command of PLA.

3.2. Eligibility criteria

3.2.1. Inclusion criteria

1. Age ≥18 and <75 years;
2. hospitalized;
3. Severe COVID-19:

    1) Laboratory confirmation of SARS-CoV-2 infection by reverse-transcription polymerase chain reaction (RT-PCR) from any diagnostic sampling source
    2) Confirmed pneumonia by chest computed tomography imaging
    3) Apply to any one of the following after onset: 1)dyspnea (RR ≥ 30 times/min), 2)finger oxygen saturation ≤ 93% in resting state, 3)arterial oxygen partial pressure (PaO2) / oxygen absorption concentration (FiO2) ≤ 300MMHG, 4)pulmonary imaging shows that the focus progress > 50% in 24-48 hours

4. Confirmed interstitial lung damage by chest computed tomography imaging.

3.2.2. Exclusion criteria

1. Pregnancy, lactation and those who are not pregnant but do not take effective contraceptives measures
2. Patients with malignant tumor, other serious systemic diseases and psychosis
3. Patients who are participating in other clinical trials
4. Inability to provide informed consent or to comply with test requirements.
5. Co-Infection of HIV, tuberculosis, influenza virus, adenovirus and other respiratory infection virus.
6. Invasive ventilation
7. Shock
8. Combined with other organ failure(need organ support)
9. Interstitial lung damage caused by other reasons (in 2 weeks)

10. The pulmonary imaging revealed the interstitial damage of lungs before the COVID-19 confirmed.

3.3. Interventions

3.3.1. Investigational medicinal product

In this study, the umbilical cord-derived mesenchymal stem cells (UC-MSCs) will be tested for treatment. The UC-MSCs are harvested from human umbilical cord. The product is almost colorless suspension, which contains 4.0×10^{7} MSCs with a volume of 100ml/bag. The placebos are with the same appearance in packaging and the suspension without MSCs. The dose of the treatment is 4.0×10^{7} (1 bag) / procedure, three procedures for every patient on day 0, day 3, and day 6 after randomization.

The administration procedure is as follows:

1) check the patient status and emergency equipment
2) verify UC-MSC packaging and patient information
3) use saline to flush the catheter before infusion
4) shake the bag gently when flocculant is notable in the bag
5) intravenous infusions in 30 minutes, press the bag gently to prevent cell clumping if necessary.
6) use 10 ml to 30 ml saline to flush the bag to ensure maximum MSC infusion
7) closely monitoring the patients' ECG, blood oxygen saturation, body temperature, pulse, skin color, respiration, and blood pressure during the entire procedure.
8) Dispose of medical waste with protection to prevent potential virus transmission

3.3.2. Standard of care (SOC)

The standard of care is following the updated guideline issued by the Chinese National Health Commission (7th edition). The principle of the treatment is supportive care in addition to infection prevention and control as necessary, including the following measures: respirational support (supplemental oxygen, noninvasive and invasive ventilation, and extracorporeal membrane oxygenation), vasopressor support, renal-replacement therapy.

Any concomitant care (medications, procedures) in this study will be recorded.

3.3.3. Intervention assignment

Experimental group: UC-MSCs administration on day 0, day 3, day 6, plus standard of care during the entire trial.
Control group: UC-MSCs placebo administration on day 0, day 3, day 6, plus standard of care during the entire trial.

Patients will be randomly assigned to either one of the treatment groups. See section 3.8 in detail.

3.3.4. Modifications of intervention

Criteria for discontinuing of the intervention:

1) Patient withdraws consent
2) Poor compliance results in incomplete administration of UC-MSCs per protocol.
3) Serious adverse events judged by the investigator that the patient is no longer safe to continue UC-MSCs treatment.
4) Any other event judged by the investigator that the patient should be withdrawn from the study

3.4. Outcomes

In this study, the primary endpoint and a part of secondary endpoints will be evaluated by the changes in high-resolution chest computed tomography and measured by a centralized imaging interpretation process.

3.4.1 Imaging Endpoints by centralized interpretation

Primary endpoint measures (centralized imaging interpretation**)**

- Change in lesion proportion (%) of full lung volume from baseline to day 28.
  Lesion proportion = lesion volume (in cm^3) / full lung volume (in cm^3)

Secondary endpoint measures (centralized imaging interpretation**)**

- Change in lesion proportion (%) of full lung volume from baseline to day 10 and 90
- Change in solid component lesion proportion (%) of full lung volume from baseline to day 10, 28 and 90.
- Change in ground-glass lesion proportion (%) of full lung volume from baseline to day 10, 28 and 90.
- Pulmonary fibrosis – related morphological features in CT scan at day 90
    a. cord-like shadow
    b. honeycomb-like shadows
    c. interlobular septal thickening
    d. intralobular interstitial thickening
    e. pleural thickening
- Lung densitometry:
    a. Change in total voxel ‘weight’ in lesion area from baseline to day 10, 28 and 90.
       voxel ‘weight’=voxel density (in HU) × voxel volume (in voxel)
    b. volumes histogram of lung density distribution (<-750, -750~-300, -300~50, >50) at day 10, 28 and 90.

3.4.2 Other secondary endpoints

1) Time to clinical improvement in 28 days.
   Clinical improvement defined as a one-point deduction from baseline in a 6 ordinal scale:

1. Not hospitalized;
2. Hospitalized, not requiring supplemental oxygen;
3. Hospitalized, requiring supplemental oxygen;
4. Hospitalized, on non-invasive ventilation or high flow oxygen devices;
5. Hospitalized, on invasive mechanical ventilation or ECMO;
6. Death.

2) Change in oxygenation index (PaO_2/FiO_2) from baseline to day 6, 10, and 28.
3) The duration of oxygen therapy (in days)
4) Change in oxygen saturation from baseline to day 6, 10 and 28
5) 6-minute walk test at day 28 and 90 (in meters)
6) Pulmonary function test at day 28 and 90
   a. maximum vital capacity (VC_{max})
   b. diffusing Capacity (DL_{CO})
7) Difference in the change of mMRC (Modified Medical Research Council) Dyspnea Scale at day 28, day 90.
8) Changes of absolute lymphocyte counts and subsets, as well as cytokine/chemokine levels from baseline to day 6, 10, 28 and 90.

3.4.3 Safety endpoints

1) Adverse events
2) Serious adverse events
3) All-cause mortality

3.4.4 Centralized imaging interpretation of endpoints based on high-resolution chest computed tomography imaging

An independent central imaging reading and adjudication committee will be set for the evaluation of the four high-resolution chest CTs of every patient (baseline, day 10, 28, and 90). Centralized imaging interpretation process will be conducted separately when all patients finish 28-day follow-up (for baseline, day 10 and 28 chest CT) and 90-day follow-up (for day-90 chest CT). The reviewers will be blinded for the patient's visit information for the 28-day review and the treatment allocation for both reviews.

The review contains a software assisted lung volumetry and densitometry procedure and a manual review procedure.

Software assisted lung volumetry and densitometry procedure

1) Automatic lung segmentation: Import raw CT images to lung densitometry software. The software will automatically conduct lung segmentation for full lung area and lesion area.
2) Independent manual correction of segmentation: Two reviewers will manually correct the lesion segmentation by the software in itk-snap platform (V 3.8.0).
3) Volumetry and densitometry result output: if the difference in lesion proportion (%) of full lung volume between the two reviewers is no more than 10%, then the volumetry and densitometry will be recorded. The final result will be the average of the two reviewers. If the

difference is more than 10%, then a third reviewer with a higher level will be introduced in the review process. The third reviewer's result will be the final result.

Lung volumetry and densitometry parameter:

Volumetry

- Full lung volume (in cm^3, segmented and measured by software)
- Lesion volume (in cm^3, segmentation manually corrected by reviewers, measured by software)
- solid component lesion volume (in cm^3, segmented and measured by software)
- ground-glass lesion volume (in cm^3, segmented and measured by software)

Densitometry (measured by software)

- total voxel 'weight' in lesion area (segmentation manually corrected by reviewers, measured by software)
  voxel 'weight'=voxel density (in HU) × voxel volume (in voxel)
- volumes histogram of lung density distribution (<-750, -750~-300, -300~50, >50, measured by software)

Manual review for pulmonary fibrosis – related morphological features

The two independent reviewers will evaluate the following pulmonary fibrosis – related morphological features in CT scan at day 90. When results are different, the result of a third reviewer with a higher level will be the final call.

a. cord-like shadow
b. honeycomb-like shadows
c. interlobular septal thickening
d. intralobular interstitial thickening
e. pleural thickening

3.5. Participant timeline

Figure**.** The schedule of enrolment, interventions, and assessments.

STUDY PERIOD	Screening	Treatments			Follow-ups			
**TIMEPOINT (DAY)**	***-7~0***	**0**	**3**	**6**	***10***	***14****	***28#***	***90***
**VISIT**	***E***	***T1***	***T2***	***T3***	***F1***	***F2***	***F3***	***F4***
**ENROLMENT:**								
**Informed consent**	X							
**Eligibility screen**	X							
Demographical characteristics	X							
Medical History	X							
**Urine pregnancy test (Women)**	X							X
**Urinalysis**	X							
**Stool analysis**	X							
**INTERVENTIONS:**								
**Allocation**		X						
Experimental Group UC-MSCs		X	X	X				
Control Group UC-MSCs Placebo		X	X	X				
Both Groups Standard of Care	→	→	→	→	→	→	→	→
**ASSESSMENTS:**								
Physical Examination	X	X	X	X	X		X	X
Chest CT	X				X		X	X
mMRC Dyspnea Scale		X		X	X		X	X
6-minute walk test							X	X
Pulmonary function (VC max and DLco)							X	X

Blood oxygen saturation	X		X	X			X	X
ECG	X						X	X
Lab Tests**	X	X	X	X	X		X	X
Adverse event	→	→	→	→	→	→	→	→
Concomitant care	→	→	→	→	→	→	→	→

* visit by phone call or phone app.
\# Time window is from day28 to day35.
** including complete blood count, liver and kidney function, electrolyte panel, blood glucose, procalcitonin (PCT), Interleukin-6 (IL-6), creatine kinase (CK), troponin, myoglobin, brain natriuretic peptide(BNP), lactate dehydrogenase, D-dimer, C-Reactive Protein (CRP), absolute lymphocyte counts and subsets (including CD4 T, CD8 T, NK, B), cytokine/chemokine levels (including IL-1β, IL-2, IL-4, IL-5, IL-6, IL-8, IL-10, IL-12P70, IL-17A, IL-17F, IL-22, TNF-α, TNF-β, IFN-γ, IL-1RA, IL-18, G-CSF, RANTES, MCP-1, IP-10, MIP-1α)

3.6. Sample size

Due to the exploratory nature of this pilot trial and limited efficacy information of treatment in COVID-19 patients, no statistical hypothesis was made. The original target sample size was set to be 45 patients, with the allocation ratio of 2:1, 30 in the UC-MSCs group, and 15 in the placebo group. The enrolment soon reached the target number because of the rapid outbreak of COVID-19 in Wuhan city. Considering minimal serious adverse events were observed, we decided to expand the sample size to gathering more information from this study. The sample size first expanded to 60, then currently set at 90 with 60 patients in the UC-MSC group, 30 patients in the placebo group in the situation that fewer new COVID-19 patients were available since the outbreak was contained in Wuhan. Decisions were made in a manner to maintain the double-blind status of this study and approved by the institutional review board.

3.7. Recruitment

Patient will be recruited from two designated hospitals for COVID-19 in Wuhan City, Hubei Province: Huoshenshan Hospital and Hubei Maternity and Child Health Care Hospital. No other recruitment strategy (e.g., advertisement) will be adopted in this study.

3.8. Allocation, blinding and product management

To ensure the cytoactive of UC-MSCs, the stem cells need to be administrated to patients in 24 hours after production and require a strict storing condition (8-12 ℃, light-sensitive). Moreover, UC-MSC packages are produced in Tianjin, which is more than 1000 kilometers away from Wuhan. To face the challenges in treatment allocation, product management, and logistics, a barcode tracking system will be introduced to this study. The system is developed upon a post-market medical product tracking system currently been used by national drug administration agencies, hospitals, and patients in China. The system will be embedded with drug tracking, management, shipping (including shipment position and storing temperature) based on unique barcodes of every bag and package. It will be connected to a web-based randomization system.

3.8.1. Allocation and concealment of allocation

The permutated-block randomization sequence is generated by a non-investigator of this study and uploaded to the randomization system. The patients will be randomly assigned to either the UC-MSC group or placebo group with a 2:1 allocation ratio stratified by sites. The concealment of the randomization sequence, block size will be ensured by the randomization system.

3.8.2. Blinding

This is a double-blind trial. Patients, investigators, and outcome assessors (independent central imaging reviewers) will all be blinded to treatment allocation. The blinding will be ensured by product making and packaging (the UC-MSC and the placebo cannot be identified by appearance

and packaging). The unique barcode, package numbers are also non-informative. To further ensure the blinding procedure, each shipping package unit will include extra bags of UC-MSCs and placebos bags, and not all products will be used.

The emergency unblinding procedure will be triggered under the circumstances when knowledge of the patient's treatment allocation is necessary for handling an emergency (e.g., when a severe adverse event is observed, the investigator needs to know the treatment group for further management of the patients). When facing such circumstances, the investigator will log into the randomization system by web or by phone app and conduct the emergency unblinding procedure online. The system will record and notify the principal investigator for all unbinding activities.

3.8.3. Product management

In this trial, all study products (UC-MSC and placebo) logistics will be enhanced by the Product Identification Authentication and Tracking System (PIATS). The system is connected to the study's randomization system. When the enrollment process is ongoing, the PIATS will ensure the study site have enough product in-store. When storing is low, it will trigger a new batch of the products' shipping process.

The entire product management process: prepare, packaging, shipping, storing, and clinical administration to patients will be conducted and traced by barcode scanners. The barcode will be unique and also non-informative to the blinded personnel. Investigators can view production information, track shipping status, revive shipment, dispense to patients using the system, and the barcode scanner.

3.9. Data collection and data management

This trial will be conducted under an infectious disease public health emergency, which makes clinical trial challenging in data collection and management. Non-healthcare personnel cannot be involved in getting direct contact with hospitalized patients with active COVID-19. Most of the work solely rely on doctors and nurses on-site, and they are already putting most of the effort on providing medical care for COVID-19 patients. There will not be additional assistance from a third party (e.g., contract research organizations) with data collection and on-site monitoring when patients are hospitalized, their work will only begin from the follow-up phase.

In this trial, most of the research data will be collected from electronic medical records (EMRs). Doctors and nurses in this study will follow a guidance to collect trial-necessary source data in combination with patients' routine medical records. All trial related EMRs will be authorized to export to an electronic source data platform to perform data integrating, standardizing and cleaning and verifying. Data derived from EMRs or non-EMR data will be manually collected by investigator (e.g. time to clinical improvement) or study coordinator.

The data of the centralized imaging interpretation will be directly exported from softwares in medical imaging workstation.

3.10. Statistical consideration

3.10.1. Statistical methods

This study is designed to be a phase II, exploratory clinical trial. We will focus on statistical description for statistical analyses. Because there are no pre-defined hypotheses made in this study, all statistical test, confidence intervals, P-value is for reference, not for inference.

Baseline characteristics will be provided using descriptive statistics as frequencies (percentage) or mean±SD.

The UC-MSCs group will be compared against the control. Both intention-to-treat (ITT) and per-protocol strategy will be adopted when analyzing primary and secondary outcomes. The full analysis set (FAS) will be considered as the primary analysis population. We will use chi-square test/fisher's exact test for binary outcomes, and t-test/Wilcoxon rank-sum test for continuous outcomes as appropriate. For comparison of changes from baseline between the two groups, an ANCOVA model considering baseline value as a covariate will be conducted in addition to direct comparison of the difference from baseline.

Adverse events, severer adverse events will be described in event frequency and proportion. Safety outcomes will be analyzed per treatment based on the safety population.

All statistical tests will be performed at a two-tailed test of $P<0.05$ statistically significant level. Analyses will be conducted using SAS software (version 9.4, NC, USA)

3.10.2. Additional analyses

All detailed analyses will be pre-specified in the statistical analysis plan. Considering the exploratory nature of this trial, additional analyses may be applied. All additional analyses which not pre-specified will be noted as post-hoc analyses.

3.10.3. Analysis population and missing data

Full Analysis Set (FAS): Including all randomized patients based on ITT principles. Only the following circumstances will be excluded: No UC-MSC or placebo are administrated after randomization.

Per-Protocol Set (PPS): Patients with major protocol violation that may affect the evaluation of primary endpoint.

Safety Set (SS): Patients with at least one dose of UC-MSC or placebo treatment

Baseline description will be performed in FAS, primary and secondary analysis will be performed in FAS and PPS population. Safety analysis will be performed in SS.

For primary analysis in FAS population, when the patient is missing a chest CT scan, the last scan's result will be carried to the missing visit. Other missing values of secondary outcomes and PP analyses will not be imputed.

3.11. Safety/harms

Adverse events (AEs) and severer adverse events (SAEs) will be monitored and recorded from the time the subject signed informed consent to the completion of the follow-ups. AEs/SAEs will be recorded in detail, including onset date, duration, severity, treatment, relation to the investigational medical product. All AEs/SAEs will be followed up until finalized (recover/relief, stable, deaths, or other explainable circumstances, e.g., lost to follow-up).

SAEs, once identified, must be taken into action and reported within 24 hours. SAEs will be reported to IRB as soon as possible.

3.12. Auditing

This study will be conducted following Good Clinical Practice (GCP). Investigators and coordinators will conduct source data verification when patients are hospitalized. Additional auditing and quality control by the site's GCP office and contract research organizations will be adopted. There will not be third party auditing in this trial during the COVID-19 outbreak, it will be available in the follow-up phase when all patients are discharged from hospital.

4. ETHICS AND DISSEMINATION

4.1. Research ethics approval

This trial's protocol and informed consent forms have been approved by the institutional review boards of the Fifth Medical Center of PLA General Hospital. Major protocol amendments, SAEs suggestion will also be reported to IRB.

4.2. Protocol amendments

Major changes, such as study objectives, study design, patient population, sample sizes, study procedures, outcomes which may impact potential benefit or harm of the patients will require a formal amendment to the protocol and will be approved by IRB.

4.3. Informed consent process

The informed consent will be obtained from eligible hospitalized COVID-19 patients or their legal family substitute (for patients incapable of making an informed decision to give consent) by the investigators. Paper-based consent forms will be used in this study.

4.4. Confidentiality

The investigators are bound to keep all patient's records that contain names or other personal identifiers confidential. All research data will be identified by a study subject ID only. Data or records shall not be used for purposes other than this clinical study.

4.5. Declaration of interests

The principle investigators declare no financial and other competing interests.

4.6. Access to data

All investigators from the steering committee will be given full access to the final data sets.

4.7. Ancillary and post-trial care

The investigators will continue to follow up patients after 90 days for safety and research purposes. Long-period results of efficacy and safety will be collected.

4.8. Dissemination policy

The study team will communicate trial results with health authorities, professionals, and patients who participated in this study. The results of this trial will be published when available.

Further publications, authorship of this study results must be reviewed by the principal investigator and sponsor, and written consent must be obtained.

Data sharing policy will be described in detail in the data sharing statement when the study result is published.

5. STUDY ADMINISTRATION

5.1. Key contacts

Central contact

Principal Investigator: Fu-Sheng Wang, MD, Ph.D. 86-10-66933332 fswang302@163.com

Investigator and coordinator: Lei Shi, MD, Ph.D. 86-10-66933333 shilei302@126.com

Study Sites:
Optical Valley Branch of Maternal and Child Hospital of Hubei Province, Wuhan, Hubei, China, 430000
Contact: Wei-Fen Xie, MD, PhD

Wuhan Huoshenshan Hospital, Wuhan, Hubei, China, 430000
Contact: Lei Shi, MD, PhD

5.2. Roles and responsibilities

5.2.1. Protocol contributors

Fu-Sheng Wang, Wei-Fen Xie, Chen Yao designed the trial.
Chen Yao developed randomization, data management, and statistical plan.
Lei Shi and Chongya Dong drafted the protocol.

5.2.2. Sponsor and Funding and Collaborators

Sponsor
The Fifth Medical Center of PLA General Hospital

Funding
1. National Key R&D Program of China (2020YFC0841900, 2020YFC0844000).
2. Innovation Groups of the National Natural Science Foundation of China (81721002).
3. National Science and Technology Major Project (2018ZX10302104-002).

Collaborators
Study sites:

Huoshenshan Hospital, Optical Valley Branch of Maternal and Child Hospital of Hubei Province, General Hospital of Central Theater Command (Follow-up only).

UC-MSC and Placebo Provider:
VCANBIO Cell & Gene Engineering Corp., Ltd, China.

Randomization system provider:
Chengdu Cims-medtech Co., Ltd, China.

Product Identification Authentication and Tracking System (PIATS) provider:
Alibaba Health Technology (China) Company Limited, China

Central imaging reading and adjudication provider:
Wuhan Union Hospital, Tongji Medical College of Huazhong University of Science and Technology, China

Electronic source data system and eCRF system provider:
Digital China Health Technologies Co., Ltd, China

Trial Monitoring/ Data management team/ Clinical Research Organization:
SciTrials Medical Technolody Ltd, China

Statistical analysis
Department of biostatistics, Peking University First Hospital, China

5.2.3. Trial committees

Steering Committee: Fu-Sheng Wang, Wei-Fen Xie, Chen Yao
Operating Committee: Xuechun Lu, Lei Shi, Hai Huang, Xiaojing Jiang, Liangliang Sun, Fanping Meng, Ming Shi, Lei Huang, Junliang Fu, Zhe Xu, Xin Yuan, Tianyi Liu, Chao Zhang, Wenjin Song, Yuanyuan Li, Ruonan Xu, Siyu Wang.
Central Imaging Reading and Adjudication Committee: Fan Yang, Lan Zhang, Ran Tao

6. References

1. Li H, Zhang D, Xu J et al. SARS-CoV-2 and viral sepsis: observations and hypotheses. *Lancet* 2020; 395: 1517–20 Published Online April 17, 2020 https://doi.org/10.1016/ S0140-6736(20)30920-X.
2. WHO. 2020. Coronavirus disease (COVID-19) situation reports https://www.who.int/emergencies/diseases/novel-coronavirus-2019/situation-reports -accessed 24rd May 2020.
3. Tang N, Li D, Wang X, Sun Z. Abnormal coagulation parameters are associated with poor prognosis in patients with novel coronavirus pneumonia. *J Thromb Haemost* 2020; published online Feb 19. DOI:10.1111/jth.14768.
4. Giamarellos-Bourboulis EJ, Netea MG, Rovina N, et al. Complex Immune Dysregulation in COVID-19 Patients with Severe Respiratory Failure. *Cell Host Microbe* 2020.
5. Xu Z, Shi L, Wang Y, Zhang J, et al. Pathological findings of COVID-19 associated with acute respiratory distress syndrome. *Lancet Respir Med* 2020; **8:** 420-422.
6. Zumla A, Hui DS, Azhar EI, Ziad A , Memish, Markus Maeurer. Reducing mortality from 2019-nCoV:

host-directed therapies should be an option. *Lancet* 2020; **395:** e35-e36.
7. Galipeau J, Sensebe L. Mesenchymal Stromal Cells: Clinical Challenges and Therapeutic Opportunities. *Cell Stem Cell* 2018; **22:** 824-33.
8. Behnke J, Kremer S, Shahzad T, et al. MSC Based Therapies-New Perspectives for the Injured Lung. *J Clin Med* 2020; 9(3).
9. Chuang H-M, Shih TE, Lu K-Y, et al. Mesenchymal Stem Cell Therapy of Pulmonary Fibrosis: Improvement with Target Combination. *Cell Transplant* 2018: 963689718787501.
10. Lazarus HM, Haynesworth SE, Gerson SL, Rosenthal NS, Caplan AI. Ex vivo expansion and subsequent infusion of human bone marrow-derived stromal progenitor cells (mesenchymal progenitor cells): implications for therapeutic use. *Bone Marrow Transplant* 1995; **16:** 557-64.
11. Jacques Galipeau andLuc Sensébé. Mesenchymal Stromal Cells: Clinical Challenges and Therapeutic Opportunities. Cell Stem Cell 2018; **22:** 824-33.
12. Ball LM, Bernardo ME, Roelofs H, et al. Multiple infusions of mesenchymal stromal cells induce sustained remission in children with steroid-refractory, grade III-IV acute graft-versus-host disease. *Br J Haematol* 2013; **163:** 501-9.
13. Wilson JG, Liu KD, Zhuo H, et al. Mesenchymal stem (stromal) cells for treatment of ARDS: a phase 1 clinical trial. *Lancet Respir Med* 2015; **3:** 24-32.
14. Matthay MA, Calfee CS, Zhuo H, et al. Treatment with allogeneic mesenchymal stromal cells for moderate to severe acute respiratory distress syndrome (START study): a randomised phase 2a safety trial. *Lancet Respir Med* 2019; **7:** 154-62.
15. Giamarellos-Bourboulis EJ, Netea MG, Rovina N, et al. Complex Immune Dysregulation in COVID-19 Patients with Severe Respiratory Failure. *Cell Host Microbe* 2020.
16. Paul Lohan, Oliver Treacy, Matthew D Griffin, et al. Anti-Donor Immune Responses Elicited by Allogeneic Mesenchymal Stem Cells and Their Extracellular Vesicles: Are We Still Learning? *Front Immunol* 2017;**8:**1626.
17. Mary Thompson, Shirley H J Mei, Dianna Wolfe 1, et al. Cell Therapy With Intravascular Administration of Mesenchymal Stromal Cells Continues to Appear Safe: An Updated Systematic Review and Meta-Analysis. *E Clinical Medicine* 2020;**19:**100249.
18. Wilson JG, Liu KD, Zhuo H, et al. Mesenchymal stem (stromal) cells for treatment of ARDS: a phase 1 clinical trial. *Lancet Respir Med* 2015; **3:** 24-32.
19. Matthay MA, Calfee CS, Zhuo H, et al. Treatment with allogeneic mesenchymal stromal cells for moderate to severe acute respiratory distress syndrome (START study): a randomised phase 2a safety trial. *Lancet Respir Med* 2019; **7:** 154-62.
20. Guoping Zheng, Lanfang Huang, Haijiang Tong, et al. Treatment of Acute Respiratory Distress Syndrome With Allogeneic Adipose-Derived Mesenchymal Stem Cells: A Randomized, Placebo-Controlled Pilot Study. *Respir Res* 2014; **15:** 39.
21. Mehta P, McAuley DF, Brown M, Sanchez E, et al. COVID-19: Consider Cytokine Storm Syndromes and Immunosuppression. *Lancet* 2020; **395:** 1033-34.
22. Dienz O, Rud JG, Eaton SM, et al. Essential role of IL-6 in protection against H1N1 influenza virus by promoting neutrophil survival in the lung. *Mucosal Immunol* 2012; **5:** 258-66.
23. Huang C, Wang Y, Li X, et al. Clinical features of patients infected with 2019 novel coronavirus in Wuhan, China. *Lancet* 2020; **395:** 497-506.
24. Fan H, Zhao G, Liu L, et al. Pre-treatment with IL-1β enhances the efficacy of MSC transplantation in DSS-induced colitis. *Cellular & Molecular Immunology* 2012; **9:** 473-81.
25. Leng Z, Zhu R, Hou W, et al. Transplantation of ACE2 Mesenchymal Stem Cells Improves the Outcome of Patients with COVID-19 Pneumonia. *Aging Dis* 2020; **11:** 216-28.

7. APPENDICES

7.1 Revision History

Version	Date	Amendment Text	Description
1.1	March 6, 2020	Sample size was expanded from 45 to 60.	The original target sample size was set to be 45 patients, with the allocation ratio of 2:1, 30 in the UC-MSCs group, and 15 in the placebo group. The enrolment soon reached the target number because of the rapid outbreak of COVID-19 in Wuhan city. Considering minimal serious adverse events were observed, we decided to expand the sample size to gathering more information from this study. The sample size first expanded to 60.
		Single infusion of MSCs was altered to the same dose (4.0*10E7 cells per time).	The number of cells in a single infusion was changed from two different grades (body weight≥70Kg, 4.0*10E7 cells per time; body weight<70Kg, 3.0*10E7 cells per time) to same grade.
2.0	March 11,2020	Primary outcome measure was changed to size of lesion area and severity of pulmonary fibrosis by chest CT.	COVID-19 is an unprecedentedly new infectious disease in the world. The distribution and disease characteristics of patients are constantly developed. We adjusted the research protocol according to actual situation of enrolled patients.
		Blood oxygen saturation, oxygenation index (PaO2/FiO2), CD4+ T cell count and cytokine level and side effects were added as secondary outcome measures.	
		Improvement time of clinical critical treatment index within 28 days, all-cause mortality on Day 28, invasive mechanical ventilation rate and incidence of nosocomial infection were deleted in secondary outcome measures part.	
		The following is added to the exclusion criteria:<br>• Interstitial lung damage caused by other reasons (in 2 weeks)	

		• The pulmonary imaging revealed the interstitial damage of lungs before the COVID-19 confirmed.	
		Maternal and Child Health Hospital of Hubei Province was added as collaborators.	
		Sample size was expanded from 60 to 90.	
		Age range was expanded to 75-years-old.	
3.0	March 23, 2020	Official Title was changed to A Multicenter, Randomized, Double-blind, Placebo-controlled Study Evaluating the Efficacy and Safety of Human Mesenchymal Stem Cells in Combination With Standard Therapy in the Treatment of COVID-19 Patients With Severe Convalescence.	
		General Hospital of Central Theater Command was added as collaborators.	The follow-up procedure will be conducted at the General Hospital of Central Theater Command of PLA.
		mMRC (Modified Medical Research Council) dyspnea scale, 6-minute walk test, maximum vital capacity (VCmax), Diffusing Capacity (DLCO) were added as secondary outcome measures.	
		Proportion of patients in each classification of clinical critical treatment index was deleted in secondary outcome measures part.	
3.1 Final	June 1,2020	Outcomes were confirmed as follows:<br>Imaging Endpoints by centralized interpretation<br>Primary endpoint measures (centralized imaging interpretation)<br>•Change in lesion proportion (%) of full lung volume from baseline to day 28.	

		Lesion proportion = lesion volume (in cm3) / full lung volume (in cm3)<br>Secondary endpoint measures (centralized imaging interpretation)<br>•Change in lesion proportion (%) of full lung volume from baseline to day 10 and 90<br>•Change in solid component lesion proportion (%) of full lung volume from baseline to day 10, 28 and 90.<br>•Change in ground-glass lesion proportion (%) of full lung volume from baseline to day 10, 28 and 90.<br>•Pulmonary fibrosis – related morphological features in CT scan at day 90<br>a. cord-like shadow<br>b. honeycomb-like shadows<br>c. interlobular septal thickening<br>d. intralobular interstitial thickening<br>e. pleural thickening<br>•Change in lung densitometry:<br>a.total voxel 'weight' in lesion area<br>voxel 'weight'=voxel density (in HU) × voxel volume (in voxel)<br>b. volumes histogram of lung density distribution (<-750, -750~-300, -300~50, >50)<br><br>3.4.2 Other secondary endpoints<br>1) Time to clinical recovery in 28 days.<br>Clinical improvement defined as a one-point deduction from baseline in a 6 ordinal scale:<br>1.Not hospitalized;<br>2.Hospitalized, not requiring supplemental oxygen;<br>3.Hospitalized, requiring supplemental oxygen;<br>4.Hospitalized, on non-invasive ventilation or high flow oxygen devices;<br>5.Hospitalized, on invasive mechanical ventilation or ECMO;<br>6.Death.	

		2)Change in oxygenation index (PaO2/FiO2) from baseline to day 6, 10, and 28.<br>3)The duration of oxygen therapy (in days)<br>4)Change in oxygen saturation from baseline to day 6, 10 and 28<br>5)Difference in 6-minute walk test at day 28 and day 90 (in meters)<br>6)Pulmonary function test at day 28 and day90<br>a. maximum vital capacity (VCmax)<br>b. diffusing Capacity (DLCO)<br>7)Difference in the change of mMRC (Modified Medical Research Council) Dyspnea Scale at day 28, day 90.<br>8)Changes of absolute lymphocyte counts and subsets, as well as cytokine/chemokine levels at baseline, day 6, day 10, day 28 and , day 90.<br><br>3.4.3 Safety endpoints<br>1) Adverse events<br>2) Serious adverse events<br>3) All-cause mortality	

7.2 mMRC (Modified Medical Research Council) Dyspnea Scale

Walking should be assessed on level ground

Dyspnea only with strenuous exercise	0
Dyspnea when hurrying or walking up a slight hill	+1
Walks slower than people of the same age because of dyspnea or has to stop for breath when walking at own pace	+2
Stops for breath after walking 100 yards (91 m) or after a few minutes	+3
Too dyspneic to leave house or breathless when dressing	+4

Data S2

A Randomized double-blind, placebo-controlled evaluation of the efficacy and safety of human mesenchymal stem cells combined with standard therapies in the treatment of patients with severe COVID-19

Statistical Analysis Plan

Version number: v1.0 2020-06-05

Sponsor: The Fifth Medical Center of PLA General Hospital

Statistical unit: Peking University Clinical Research Institute

Signature Page

The authors:

Yu Yongpei, statistician

Signature Date

Report reviewer, the person in charge of quality control:

Yan Xiaoyan, Senior Statistician

Signature Date

Table of Contents

1. Table of Abbreviations 1
2. Research title 1
3. Research objectives 2
4. Trial design 2
    4.1 Study design 2
    4.2 Study intervention 2
    4.3 Randomization and blinding 2
    4.4 Research schedule 3
    4.5 Inclusion criteria 4
        4.5.1 Inclusion criteria 4
        4.5.2 Exclusion criteria 4
        4.5.3 Withdrawal criteria 5
        4.5.4 Termination criteria 5
        4.5.5 Complete research 5
        4.5.6 Withdraw from research 6
5. Data management 6
    5.1 Creating database 6
    5.2 Data input 6
6. Outcomes 6
    6.1 Primary endpoint measures 6
    6.2 Secondary endpoint measures 6
    6.3 Safety endpoints 7
7. Statistical Analysis 7
    7.1 Analysis data set 7
        7.1.1 Full Analysis Set (FAS) 7
        7.1.2 Per Protocol Set (PPS) 7
        7.1.3 Safety Set (SS) 7
    7.2 Missing data processing 7
    7.3 Statistical Methods 7
        7.3.1 Principles of Statistical Analysis 7
        7.3.2 Completion status and demographic analysis 8
        7.3.3 Baseline analyses 8
        7.3.4 Primary efficacy meansures 9
        7.3.5 Secondary efficacy measures 9
        7.3.6 Safety analysis 10
8. Explanation of statistical analysis results 10

1. Table of Abbreviations

Abbreviations	Definition
AE	Adverse event
ALT	Alanine aminotransferase
AST	Aspartate aminotransferase
BMI	Body mass index
BUN	Blood urea nitrogen
CI	Confidence interval
CK	Creatine kinase
CMH	Cochran-Mantel-Haenszel test
Cr	Creatinine
CRC	Clinical research coordinator
CRF	Case report form
CRO	Contract research organization
CRP	C-reactive protein
CT	Computed tomography
CTA	Clinical trial agreement
DMC	Data Monitoring Committee
EC	Ethics committee
EMR	Electronic medical record
F	F statistic (result of covariance analysis)
FAS	Full analysis set
FiO2	Fraction of inspiration O2 (oxygen absorption concentration)
GCP	Good clinical practices
ICF	Informed consent form
IL-6	Interleukin-6
ITT	Intent-to-treat analysis
IWRS	Interactive Web Response System (the central randomization system)
LOCF	Last observation carried forward
P	P value
PI	Principle investigator
PPS	Per-protocol set
RR	Breath rate
SAE	Serious adverse event
SD	Standard deviation
SOC	Standard of care
SOP	Standard operating procedures
SS	Safety data set
TEAE	Treatment emergent adverse event
UC-MSC	Umbilical cord mesenchymal stem cells

2. Research title

A randomized double-blind, placebo-controlled evaluation of the efficacy and safety of human mesenchymal stem cells combined with standard therapies in the treatment of patients with severe COVID-19

3. Research objectives

To evaluate the efficacy and safety of umbilical cord-derived human mesenchymal stem cells in the treatment of severe COVID-19 patients.

4. Trial design

4.1 Study design

This study is a multi-center, prospective, randomized double-blind, placebo-controlled clinical trial. The overall study design is demonstrated in the figure below:

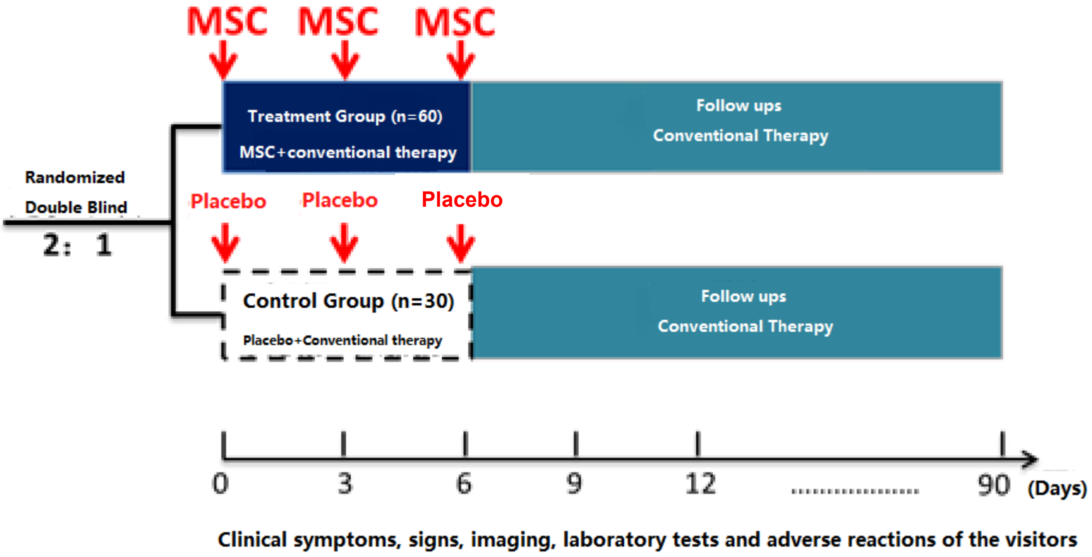

4.2 Study intervention

The allocation ratio is 2:1 in the treatment group comparing to the control group.

The treatment group (60 cases): standard treatment + human umbilical cord mesenchymal stem cell (UC-MSC) treatment (intravenous injection once every 3 days for a total of 3 times (starting from day 0), the dose of each treatment is 4.0×10^7 MSCs with a volume of 100ml).

The control group (30 cases): standard treatment plan + placebo.

Standard treatment (SOC) will be implemented according to the “Chinese Clinical Guidance for COVID-19 Pneumonia Diagnosis and Treatment (7th edition)” compiled by the National Health Commission of China.

4.3 Randomization and blinding

In order to reduce the possible selection bias and curative effect evaluation bias from investigator and subjects, this study will adopt a randomized double-blind, placebo-controlled design. Since all the patients would receive current standard care, the study has embedded patients’ interests in the design.

This study intends to use the CIMS Interactive Web Response System (IWRS), which will be docked with the "Mashangfangxin" drug blinding system by Alibaba Health. Generally, in preventing the investigators, clinical

teams or others relevant personnel from accessing the trial data, the Alibaba health system will remain inaccessible until the trial is completed. However, should circumstances arise, the Alibaba Health system also allows the emergency unblinding of individual subjects for the investigator.

To enable double-blinding, the medication of interest (the UC-MSC intravenous injections) must go through a scanning device that records the unique barcode on the medication package before use. Normally, unblinding is not allowed until all subjects have completed the study and the database is locked. Unblinding is allowed, however, when it is necessary to know the subject's treatment status to take special emergency treatment or actions. At this time, the investigator can log in to the IWRS to gather information on the subject in the emergent situation. The investigator is advised to contact the project team as much as possible before the unblinding and discuss the details. The phone of the project team or its designated personnel will be kept open 24 hours a day, 7 days a week. If the unblinding has been done, the project team must be notified as soon as possible. The date, time and reason for unblinding must be recorded in the relevant chapters and original documents of the IWRS, as well as on the electronic Case Report Form (eCRF). The unblinding document received from IWRS must be securely stored with the subject's original document.

4.4 Research schedule

STUDY PERIOD	Screening	Treatments			Follow-ups			
Time points	-7~0 days	0 days	3 days	6 days	10 days	14~21 days	28~35 days	90 days
Candidate Enrollment Screening Form	√							
Sign informed consent	√							
Demographic data	√							
Vital signs, physical examination	√	√	√	√	√		√	√
Past medical history	√							
Infusion record		√	√	√				
Blood routine	√			√	√		√	√
Procalcitonin	√			√	√		√	√
6-minute walk test							√	√
IL-6	√			√	√		√	√
Liver and kidney function, electrolytes, blood sugar	√			√	√		√	√
CK, LACTIC ACID	√			√	√		√	√
D DIMER	√			√	√		√	√

Cardiac Function	√			√	√		√	√
CRP	√			√	√		√	√
ECG	√						√	√
Chest CT	√				√		√	√
mMRC dyspnea score	√	√		√	√		√	√
Forced vital capacity (VC max)							√	√
Lung diffusion function (DL co)							√	√
Lymphocyte subsets	√			√	√		√	√
Blood gas analysis	√			√	√		√	√
Urine routine	√							
Stool routine	√							
Adverse events and concomitant medication	√	√	√	√	√	√	√	√
Telephone follow-up						√		

4.5 Inclusion criteria

4.5.1 Inclusion criteria

(1) Age ≥18 and <75 years;

(2) Hospitalized;

(3) Severe COVID-19:

1) Laboratory confirmation of SARS-CoV-2 infection by reverse-transcription polymerase chain reaction (RT-PCR) from any diagnostic sampling source
2) Confirmed pneumonia by chest computed tomography imaging
3) Apply to any one of the following after onset: 1)dyspnea (RR ≥ 30 times/min), 2)finger oxygen saturation ≤ 93% in resting state, 3)arterial oxygen partial pressure (PaO2) / oxygen absorption concentration (FiO2) ≤ 300MMHG, 4)pulmonary imaging shows that the focus progress > 50% in 24-48 hours

(4) Confirmed interstitial lung damage by chest computed tomography imaging.

4.5.2 Exclusion criteria

Subjects who meet any one of the following conditions will not be selected for the trial:

(1) Pregnancy or lactation;

(2) Patients with malignant tumor, other serious systemic diseases, or psychosis;

(3) Patients who are participating in other clinical trials;

(4) There was evidence of drug addiction within one year before joining the study;

(5) Unable or unwilling to sign the informed consent or comply with test requirements;

(6) Co-Infection of HIV, tuberculosis, influenza virus, adenovirus, or other respiratory infection viruses;

(7) Interstitial lung damage caused by other reasons (in 2 weeks);

(8) The pulmonary imaging revealed the interstitial damage of lungs before the COVID-19 confirmed;

(9) Mild, normal, or critical COVID-19 patients.

4.5.3 Withdrawal criteria

Those who meet the following criteria will withdraw from the trial:

(1) Subjects withdraw informed consent, etc.;

(2) Poor compliance results in incomplete administration of UC-MSCs per protocol.

(3) During the treatment, if the subject experienced certain comorbidities and complications, AE and SAE or special physiological changes, the investigator may decide that it was not suitable to continue the treatment. Any grade 4 AE or laboratory abnormalities related to UC-MSCs treatment, it is up to the investigator to continue the treatment of any other AE, laboratory abnormalities or concomitant diseases that are not good to the subject;

(4) Any other event judged by the investigator that the patient should be withdrawn from the study.

If a subject withdraws from the study due to AE or abnormal laboratory test results, this important special event and the test results should be recorded in the medical record.

The reasons for the dropped cases should be recorded in detail, the safety and efficacy data of the subject should be obtained as much as possible, and all the end-of-study evaluations should be carried out with the consent and compliance of the subject. All case data should be kept complete for future reference.

4.5.4 Termination criteria

The trial should be terminated if the following criteria are met:

(1) Serious safety problems occurred during the trial;

(2) The unit in charge of clinical research requests to terminate the trial (such as funding reasons, management reasons, etc.);

(3) The ethics committee or the administrative department request the termination of the trial;

(4) As the epidemic subsided, it is difficult to enroll new cases.

4.5.5 Complete research

It is expected that 90 subjects will be enrolled. Subjects will be deemed to have completed the trial after completing all the treatments and all contents specified during the follow-up period (from the screening period to the last follow-up) according to the trial protocol.

4.5.6 **Withdraw from research**

The subjects can withdraw from the study at any time during the study for any reason (special or non-special) without being unfairly treated. Subjects will not be restricted in their treatment and may continue to receive conventional treatment or other suitable treatment methods.

For subjects who are deemed ineligible in the screening period, the check items in the screening period should be stopped, and the reason for the screening failure should be recorded on the study completion/termination page. If the screened subjects withdraw from the trial, they should try to complete the check items in the current visit. And the reason for withdrawing from the study is recorded on the study completion/termination page after the study is completed.

5. Data management

5.1 Creating database

A database system project corresponding to the trial protocol will be created. The logical conditions are put in for the relevant items. The database preparation will be completed after the database passed the verification test.

5.2 Data input

The staff responsible for the data input will first receive training on the use of the database system. The trained staff will synchronize the data entry. A data administrator will review the data entries and produce a query list for the potentially problematic entries. The data entry staff will then check the original data records to verify or revise the problematic entries. Data administrators will supervise the progress of data entry and verify the data regularly during the project to ensure data quality.

6. Outcomes

6.1 Primary endpoint measures

Primary endpoint measure: changes in the high-resolution CT imaging: changes in the size, location and nature of the lesions of the lung (such as strip-like, ground-glass, grid-like, solid component, etc.).

Imaging evaluation method: Four CT images (screening period, follow-up period of 10 days, 28-35 days, and 90 days) were evaluated by the independent imaging evaluation committee of the study center.

6.2 Secondary endpoint measures

Secondary endpoints:

(1) Proportion of patients in each category on the 6-point scale at 7, 14, 28, and 90 days after randomization

(2) Blood gas analysis and oxygenation index;

(3) Duration of oxygen therapy (days);

(4) Finger pulse oxygen in resting state;

(5)6-minute walking experiment (before discharge);

(6) Lung function;

(7) mMRC dyspnea score;

(8) Features of immunological changes (changes in T lymphocytes and inflammatory factors, etc.);

6.3 Safety endpoints

No formal hypothesis testing is performed for safety analysis. Descriptive statistics will be used to summarize safety events. Safety evaluation includes the following:

- Adverse events

Collect the frequency and nature of postoperative adverse events, and evaluate the safety.

7. Statistical Analysis

7.1 Analysis data set

7.1.1 Full Analysis Set (FAS)

On the basis of the ITT principle, all randomized subjects are included as much as possible. Only the following subjects who have been randomized are excluded: violation of important core inclusion criteria; subjects not receiving treatment with experimental drugs; there are no observed data after randomization.

The FAS population is the main group of efficacy evaluation in this study.

7.1.2 Per Protocol Set (PPS)

On the basis of the FAS population, exclude subjects with protocol violations that may affect the efficacy evaluation.

The PPS population is the secondary population for the efficacy evaluation of this study.

7.1.3 Safety Set (SS)

Including all patients who received at least 1 study drug treatment.

7.2 Missing data processing

Last Observation Carry Forward (LOCF) is used for the absence of major efficacy indicators in the FAS population. The missing data on demographic information, secondary efficacy indicators, and safety indicators are not processed.

7.3 Statistical Methods

7.3.1 Principles of Statistical Analysis

The software used for statistical analysis is SAS 9.4 or above. The sample size calculation uses PASS13 software. The main and secondary outcomes are analyzed using the full analysis set and the per protocol set, and the safety data set is used to analyze safety indicators.

Unless otherwise specified, all significance tests and hypothesis tests use two-sided tests at the 5% significance level. Where appropriate, the obtained p-value will be quoted and a 95% two-sided confidence interval will be generated. All The p-value will retain three decimal places. In all tables, p-values less than 0.001

will be expressed as "<0.001".

Continuous variables will be summarized with summary statistics such as mean, standard deviation, median, minimum, maximum, lower quartile (Q1), upper quartile (Q3), etc. Categorical and ordinal variables will be summarized in terms of frequency and percentage.

The comparison between the two groups of general conditions will be analyzed by appropriate methods based on the type of indicators. The two-sample t test (if the data is normally distributed) or the Wilcoxon rank sum test (if the data is not normally distributed) will be used for the comparison of the two continuous indicators. Categorical data will be analyzed by the Chi-square test or exact probability method (if Chi-square test is not applicable); the grade data will be analyzed by the Wilcoxon rank sum test or the CMH test.

7.3.2 **Completion status and demographic analysis**

The number of people in two groups in the data set, the distribution of cases in each center, and the dropout rate are statistically described. A detailed list of reasons for data incompleteness will be compiled. The analysis of the completion of the trial will be based on all randomized subjects.

Statistical description and comparison between groups of baseline demographic information (including but not limited to age, gender, ethnicity, medical history, and medication history) are performed. Summary statistics will be utilized according to the nature of the variable (continuous variable or categorical variable).

The demographic characteristics (age, height, vital signs, etc.), medical history, and medication history of the patients are statistically described, and the age, height, weight, etc. of the two groups are compared to measure the comparability of the two groups. When the characteristics are statistically different, the analysis of the primary efficacy indicators needs to consider to adjust the statistically different baseline factors.

The analysis of baseline demographic information and vital signs will be based on the FAS.

7.3.3 **Baseline analyses**

The baseline evaluation was performed on the FAS and PPS sets. The baseline analysis used the observation value that is the most recent to the treatment start time during the screening period and the day 0 of the treatment period.

The baseline 6-point scale and the mMRC dyspnea score are statistically described as qualitative indicators, and the Wilcoxon rank sum test is used for comparison between groups.

Baseline blood gas analysis, finger pulse oxygen at rest (referring to oxygen saturation), lung function (forced vital capacity, lung diffusion function, etc.), lymphocyte subsets, IL-6, d-dimer and CRP Quantitative indicators are statistically described and compared between groups.

7.3.4 Primary efficacy meansures

The primary efficacy evaluation is performed on the FAS and PPS.

The primary efficacy measure of this study is the changes in high-resolution CT imaging. The relative changes from baseline between the two groups at 28 days after treatment are statistically described as quantitative indicators, and the t-test or the Wilcoxon rank sum test is used for comparison between groups. At the same time, the covariance model, including center and baseline as covariables, is constructed. The least-square mean (LSmean), inter-group difference and 95% confidence interval of the two groups were calculated.

7.3.5 Secondary efficacy measures

The secondary efficacy evaluation is performed on the FAS and PPS.

(1) Proportion of patients in each category of the 6-point scale

According to the classification index, the 6-point scale for each follow-up is statistically described, and the Wilcoxon rank test is used for comparison between groups.

(2) Blood gas analysis and oxygenation index

The blood gas analysis indicators of each follow-up are statistically described and compared as quantitative indicators between groups.

(3) Duration of oxygen therapy (days)

The duration of oxygen therapy is statistically described and compared as quantitative indicators between groups.

(4) Finger pulse oxygen at rest

The finger pulse oxygen is statistically described and compared as quantitative indicators between groups in each follow-up resting state.

(5) 6-minute walk experiment

The follow-up 6-minute walk experiment is statistically described and compared as quantitative indicators between groups.

(6) Lung function

The pulmonary function indicators of each follow-up are statistically described and compared as quantitative indicators between groups.

(7) mMRC dyspnea score

The 6-point scale for each follow-up is statistically described as qualitative indicators, and the Wilcoxon rank test was used for comparison between groups.

(8) Features of immunological changes (changes in T lymphocytes and inflammatory factors, etc.)

The immunological indicators such as changes in T lymphocytes and inflammatory factors at each follow-up and the changes from baseline are statistically described and compared as quantitative indicators between groups.

7.3.6 Safety analysis

The safety evaluation includes adverse events, laboratory examinations, electrocardiogram examinations and combined medications. The safety evaluation is carried out on the SS set.

1) Adverse events:

Describe the total adverse events, adverse events that led to the dropout, adverse reactions, and serious adverse events in the two groups will be reported respectively. The number of various events and the number of people who are associated with the events will be counted. The percentage of the people suffered from the adverse events in each group will be compared using the Fisher exact probability method.

2) Laboratory examination and ECG examination

The laboratory tests of clinical significance and the ECG examination at each measurement time point before and after the treatment is statistically described.

8. Explanation of statistical analysis results

Data S3

Six-category scale for COVID-19 patients

**Six-category scale for COVID-19 patients**	
1	discharge (alive)
2	hospital admission, not requiring supplemental oxygen
3	hospital admission, requiring supplemental oxygen
4	hospital admission, requiring high-flow nasal cannula or non-invasive mechanical ventilation
5	hospital admission, requiring extracorporeal membrane oxygenation or invasive mechanical ventilation
6	death

Data S4

Normal Range of Lab Test

	Normal range
White blood cell count (10^9/L)	3.5-9.5
Lymphocyte count (10^9/L)	1.1-3.2
CD4 T cells (/µl)	550-1440
CD8 T cells (/µl)	320-1250
B cells (/µl)	90-560
NK cells (/µl)	150-1100
Neutrophil count (10^9/L)	1.8-6.3
Platelet count (10^9/L)	125-350
Haemoglobin (g/L）	316-354
D-dimer (mg/L)	0-0.55
CRP (mg/L)	0-4
IL-6 (pg/ml)	0-11.09
IL-8 (pg/ml)	0-15.71
IFN-γ (pg/ml)	0-4.43
IL-1Ra (pg/ml)	131.6-340.01
IL-18 (pg/ml)	57.8-364.4
MCP-1 (pg/ml)	0-36.66
MIP-1α (pg/ml)	0-18.99
IP-10 (pg/ml)	0-23.47

Data S5

Data Sharing Statement

Data

Data available: Yes.

Data types: Deidentified participant data.

How to access data: Contact Fu-Sheng Wang at fswang302@163.com

When available: With publication

Supporting documents

Document types: None

Additional information

Who can access the data: researchers whose proposed use of the data has been approved

Types of analysis: Such requests are at the Academic Committee's discretion and dependent on the nature of the request, the merit of the research proposed, availability of the data and the intended use of the data.

Mechanisms of data availability: After approval from the Academic Committee, this trial data can be shared with qualifying researchers who submit a proposal with a valuable research question. A contract should be signed.

Any additional restrictions: NA.
